# The tale of young blood rejuvenating the old

**DOI:** 10.18632/aging.205069

**Published:** 2023-09-13

**Authors:** Xiaogang Chu, Raghavan Pillai Raju

**Affiliations:** 1 Department of Pharmacology and Toxicology, Medical College of Georgia, Augusta University, Augusta, GA 30907, USA

**Keywords:** aging, extracellular vesicles, juvenile factors, oxidative stress, Nrf2

The tale of the fountain of youth has been around at least from the time of Herodotus in the 5th century BCE. Since then, there have been several historical references of attempts/methods to restore youthfulness or slow aging, including the journey of Juan Ponce de Leon in the 16th century to Florida in search of the Fountain of Youth. The empirical evidence for the rejuvenating effect of young blood has its basis in the parabiosis experiment described by Paul Bert in 1864. This effect was not tested in the context of aging until the late 1950s when Clive McCay et al. (1956) made parabiotic pairs between young and old rats. McCay et al. intended to find “whether a young animal has substances circulating in its blood that can rejuvenate the old or vice versa” [1]. This question was later followed up by Rando and colleagues at Stanford University using heterochronic parabiosis. Their work demonstrated that the aging systemic environment is less effective in maintaining the myogenic fate of muscle stem cells and observed improved regeneration of the stem cells in older parabionts [2].

When we subjected young (5–6 weeks), adult (3–4 months), and aged (23–26 months) mice to hemorrhagic shock injury (HI), we found that the young mice survived for a more extended period, compared to others, in the absence of fluid resuscitation. The result suggested a survival advantage for the young mice, possibly indicating a maturation-dependent decline in the ability to survive following the acute injury [3]. Based on this observation and the results of previous studies using parabiosis, we hypothesized that the blood of young mice might have molecular signatures of their resilience to HI. Therefore, we sought to determine whether factors derived from juvenile plasma can improve outcomes following HI in older mice. When extracellular vesicle (EV)-enriched plasma fraction isolated from the young (Y-EVs) or aged mice (A-EVs) was administered to old mice following HI, in the absence of fluid resuscitation, the mice that received Y-EVs survived for a longer duration [3]. Furthermore, the mice that received the Y-EVs showed better mitochondrial function, indicating improved cellular energetics in the old recipients. EVs are a group of membrane nanoparticles released from the cells. They carry a variety of proteins, lipids, and nucleic acids and are present in all body fluids, including circulating blood. Plasma EVs likely represent secretions from all cell types in the body [4].

Systemic inflammation and enhanced oxidative stress are characteristics of HI. Y-EVs treatment reduced inflammation and alleviated oxidative stress in the old mice, after HI. Interestingly, the activity of Nrf2, a “master regulator” in antioxidant response regulation, was significantly enhanced in the liver following Y-EVs treatment. The transcription factor Nrf2 is crucial in antioxidant responses and redox homeostasis. Nrf2 modulates the expression of hundreds of genes, regulating antioxidants, cytoprotection, anti-inflammatory processes, tissue remodeling, and carcinogenesis [5]. In addition, Nrf2 signaling plays an essential role in regulating the cellular response in association with several other signaling pathways that control fundamental cellular processes, such as apoptosis, proliferation, migration, and angiogenesis.

Though our studies demonstrated an antioxidant effect for Y-EVs, the specific factor(s) imparting the effect in the Y-EVs remains unknown. The maturational dependence of these factors that differentiate Y-EVs from EVs isolated from older ages also needs to be established. A recent study demonstrated that EVs from young animals improved aged cell bioenergetics and skeletal muscle regeneration in a Klotho mRNA-dependent manner [6]. Another report showed GSTM2 activity in small EVs isolated from fibroblasts of young donors. These small EVs reversed the accumulation of reactive oxygen species (ROS), prevented lipid peroxidation, and ameliorated senescence-associated tissue damage [7]. Furthermore, it is known that exosomal-Nrf2 can modulate oxidative stress and induce tissue repair and regeneration.

Emerging experimental evidence suggests that aging is associated with a decrease in “pro-youthful” factors and an increase in “pro-aging” factors [8]. The mechanisms underlying the rejuvenating effects of soluble factors in the “young” blood are mainly unknown. Moreover, the question of whether these factors share molecular identities with those observed in stem cells also remains unanswered. Further research is needed to define the identity and mechanistic processes of juvenile protective factors and their relationship to organismal maturation, organ cross-talk, and aging.
